# Aluminum-Centered Tetrahedron-Octahedron Transition in Advancing Al-Sb-Te Phase Change Properties

**DOI:** 10.1038/srep08548

**Published:** 2015-02-24

**Authors:** Mengjiao Xia, Keyuan Ding, Feng Rao, Xianbin Li, Liangcai Wu, Zhitang Song

**Affiliations:** 1 State Key Laboratory of Functional Materials for Informatics, Shanghai Institute of Micro-system and Information Technology, Chinese Academy of Sciences, Shanghai 200050, China; 2 State Key Laboratory on Integrated Optoelectronics, College of Electronic Science and Engineering, Jilin University, Changchun 130012, China; 3 Graduate University of the Chinese Academy of Sciences, Beijing 100080, China

## Abstract

Group IIIA elements, Al, Ga, or In, etc., doped Sb-Te materials have proven good phase change properties, especially the superior data retention ability over popular Ge_2_Sb_2_Te_5_, while their phase transition mechanisms are rarely investigated. In this paper, aiming at the phase transition of Al-Sb-Te materials, we reveal a dominant rule of local structure changes around the Al atoms based on *ab initio* simulations and nuclear magnetic resonance evidences. By comparing the local chemical environments around Al atoms in respective amorphous and crystalline Al-Sb-Te phases, we believe that Al-centered motifs undergo reversible tetrahedron-octahedron reconfigurations in phase transition process. Such Al-centered local structure rearrangements significantly enhance thermal stability of amorphous phase compared to that of undoped Sb-Te materials, and facilitate a low-energy amorphization due to the weak links among Al-centered and Sb-centered octahedrons. Our studies may provide a useful reference to further understand the underlying physics and optimize performances of all IIIA metal doped Sb-Te phase change materials, prompting the development of NOR/NAND Flash-like phase change memory technology.

Phase change memory (PCM) has been considered to be the most promising candidate for the next generation nonvolatile memory due to its prominent advantages, such as fast switching speed, good endurance, good scalability, and compatibility with complementary metal oxide semiconductor (CMOS) technology^1^. Electrical pulse with nanosecond width is able to induce the reversible phase transition between the amorphous (highly resistive, Reset) and the crystalline (lowly resistive, Set) states. The high-to-low resistance contrast is used to store the information data^2^.

As the flagship phase change material system, Ge-Sb-Te alloys can be regarded as the combination of GeTe (GT) and Sb_2_Te_3_ (ST) along the pseudobinary tie-line. The two binary construction units have small lattice parameter deviations (2.2% for hexagonal lattice and 1.0% for rocksalt lattice)^3^. In the rocksalt Ge_2_Sb_2_Te_5_ (GST), Ge-centered and Sb-centered octahedrons resemble each other because of their resonant bonds by sharing *p* valence electrons between Ge (Sb) and six vertex Te atoms^4^. Such “twins-like” construction units, from atomic and electronic configurations perspectives, facilitate the homogeneous and meanwhile stable phase composition for both amorphous (a-) and crystalline (c-) GST even after more than 100 million times repetitive Reset-Set operations. The rationale to dope Ge element into a-ST also lies in that the spontaneous crystallization of pure a-ST at low temperature cannot guarantee the long-term stability of a-phase^5^,^6^. The a-ST is swiftly crystallized via reordering its defective or destructive Sb-centered octahedrons^7^. While crystallization of Ge doped a-ST needs to rearrange the majority of Ge atoms from tetrahedral to octahedral sites via overcoming ~2.3 eV energy barrier^8^. Thereby the rigidity of a-phase network is significantly enhanced, restraining the easy structure reordering.

Many efforts, imitating the construction principles of GST or Ge doped ST alloys, have been made to design homogeneous M-Sb-Te (M = dopant element) alloys. Namely, it attempts to design superior new phase change materials by devising “twins-like” M-centered and Sb-centered octahedrons. They are expected to coexist in a uniform crystal lattice, since improving uniformity is a crucial factor for prolonging PCM cyclability.

For instance, if M is the transition metal without *p* valence electrons in its outer shell, e. g. Ti, it covalently bonds (non-resonant bonds) with surrounding six Te atoms in an octahedral configuration. Such rigid atomic motif changes little during phase transition^9^. Indeed the analogous Ti-centered and Sb-centered octahedrons (~11% hexagonal lattice parameter mismatch between TiTe_2_ and ST) still favor stacking in a single uniform lattice^9^,^10^. However, the major part of structure change in Ti-Sb-Te (TST) is argued to be the order-disorder of Sb-centered motifs surrounding the robust Ti-centered octahedrons, which is qualitatively different from that of GST^9^.

On the other hand, group IIIA metals like Al^11^,^12^,^13^,^14^,^15^, Ga^16^,^17^,^18^, and In^19^,^20^ with one *p* valence electron have also been successfully incorporated in M-Sb-Te alloys. In comparison with Ge (resonant bonding with Te) in c-GST and Ti (covalent bonding with Te) in c-TST, IIIA cation M may introduce a sort of in-between electronic configuration to c-M-Sb-Te alloys. Still it may meet the requirement of forming harmonious lattice structure consisted by resembled M-centered and Sb-centered octahedrons. Despite the already proved good performances of IIIA M-Sb-Te PCM devices, the basic phase transition mechanism of IIIA M-Sb-Te is rarely studied.

Our previous studies in Al-Sb-Te materials have already demonstrated PCM performance improvements over the GST based one with the same device size, including 110 ~ 124°C data retention (85 ~ 90°C for GST)^12^,^13^, 5 ~ 10 ns Set-Reset speed (~30 ns for GST)^13^,^14^, and >60% reduced Reset energy^14^. Hence in the present work, we set Al-Sb-Te as an example to discuss its possible phase transition mechanism and extend to other IIIA M-Sb-Te materials. To elucidate the role of Al atoms in the transition process, *ab initio* simulations and nuclear magnetic resonance (NMR) measurements were carried out to study the chemical environment change of Al atoms in a- and c-Al-Sb-Te phases. A reversible Al-centered tetrahedron-octahedron transition is believed to play a major role in improving performances of Al-Sb-Te based PCM devices.

Figure 1 shows the X-ray diffraction (XRD) results of crystallized ST, Al_0.36_Sb_2_Te_3_ (Al_0.36_ST), and Al_0.69_Sb_2_Te_3_ (Al_0.69_ST) films annealed at 300°C for 3 min. Si (2 1 1) peak of the single crystal Si substrate is used for alignment to get a precise position of other diffraction peaks. All the films show rhombohedral structure (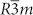 space group) with no separated Te, Al-Sb, or Al-Te phase observed, which indicates crystalline Al doped ST (Al_x_ST) can have a homogeneous phase^11^,^12^. The diffraction peaks of c-Al_x_ST are broader and weaker than those of c-ST, implying an obvious inhibition of grain growth after Al doping. As shown in the inset of Figure 1, the (0 1 5) peak positions gradually shift to higher 2 theta (2*θ*) as Al content increases (28.13° for ST, 28.16° for Al_0.36_ST, and 28.28° for Al_0.69_ST). According to Bragg's diffraction law: 2*d*sin*θ* = nλ, different values of *θ* (the angle between the incident ray and the scattering crystal planes) correspond to varied values of *d* (interplanar spacing), and reflect the change of lattice parameters. The *d* shortening between (0 1 5) planes indicates the probability of Al atoms entering the ST lattice since Al has smaller atomic radius than those of Sb and Te, which may also generate extra lattice stress between the crystal planes. It shall be correlated to the discrepancies between local chemical environments before and after Al replacements.

Rather than modelling the exact experimental composition, we used *ab initio* simulation to calculate the formation energy (*E^f^*) of a single Al atom at various lattice sites in a 60-atom c-ST supercell, so as to examine the effects of Al introducing and compare the relative stability among different Al occupancies. The *E^f^* of Al is given by^21^:
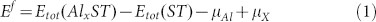
where *E_tot_* is the total energy per supercell with or without Al. *μ_Al_* is the chemical potential of Al. *μ_X_* is the chemical potential of (Sb, Te), or 0 for *X* = Al_Sb_ (Al replacing Sb), Al_Te_ (Al replacing Te), or Al_i_ (Al interstitial). There are 5 possible sites for Al in the ST lattice. The most stable site (the lowest *E^f^* = −0.35 eV) is to replace Sb (Al_Sb_) with six Te nearest neighbors. To identify the bonding chemistry between Al and Te, Figure 2 shows the charge density difference (CDD)^22^ of the relaxed structure of the Al_Sb_ model. In the Sb-centered octahedron (Figure 2b), there are three strong bonds binding Sb with 3-coordinated Te atoms, while three weak bonds are formed among Sb and other 6-coordinated Te atoms. In contrast, in the Al-centered one (Figure 2c), all the six bonds between Al and Te are relatively stronger with noticeable charge accumulation at the bond center. The tighter Al-Te bonds have shorter lengths than those of Sb-Te bonds, as shown in Table 1. To compensate for the strong Al-Te bonds, three Te-Sb bonds (in the opposite bonding direction of Al-Te) in the adjacent layer without the Al are significantly elongated and weakened, as indicated in Figure 2a by dashed lines. Note that the elongated Te-Sb bonds in c-Al_x_ST result in a larger lattice constant *c*, decreasing the interplanar distance of (0 1 5) planes (3.218 Å for c-ST and 3.204 Å for c-Al_x_ST), as shown in Figure 3. It is consistent with our analyses on the XRD results in Figure 1 and further confirms that Al atoms can occupy the Sb positions in c-ST lattice. In addition, such weakened Te-Sb bonds can be easily deformed to accommodate the lattice mismatch and relieve the tensile stress brought by the Al-centered octahedron. During amorphization, these fragile Te-Sb bonds are easily to be ruptured to break the long-range order, leading to a low-energy Reset operation.

To be noted that, in comparison with Al-Te bonds, the even stronger Ti-Te bonds in c-TST break all nine Te-Sb bonds in the adjacent layer without the Ti^9^. One may notice that the CDD on Al-Te bond is slightly weaker than that of Ti-Te (covalent) bond in Ti-centered octahedron^9^. Unlike the 3*d*^2^4*s*^2^ electronic configuration of Ti, the only *p* valence electron of Al (3*s*^2^3*p*^1^) may account for the less covalent components of Al-Te bond. Namely, a small portion of Al 3*p*-Te 5*p* bonding contributes resonant component to the Al-Te bond. This is also found from the Ga-Te and In-Te bonds in the quintuple layered (c-ST-like) lattice^23^. That is, cation M with more *p* valence electrons (larger *p-p* bonding component in M-Te bond) shall reduce the rigidity of M-centered octahedron and ease the local structure distortion to surrounding c-ST lattice^22^.

Figure 4 shows the calculated atomic structure of a-Al_0.36_ST by using the melt-quench molecular dynamics (MD) technique. The average coordination number (CN) and the peak of bond angle distribution (BAD) of Al are 4.08 and 108° in a-Al_0.36_ST, respectively, as shown in Table 2, which agrees well with the standard tetrahedral configuration (CN = 4 and BAD peak = 109°). The BAD peaks for Sb and Te are 92° and 91°, and the CNs are 3.74 and 2.82, respectively, both demonstrating the possible configuration of defective octahedrons^7^. The polyhedrons in Figure 4 highlight the Al-centered tetrahedrons (mostly Al-Te_4_) in a-Al_0.36_ST. One can also find a few Al-Sb “wrong bonds” from Sb-Al-Te_3_ tetrahedrons. Our previous X-ray photoelectron spectroscopy analyses already confirmed the existence of Al-Sb and Al-Te bonds in a-Al_x_ST^12^.

Figure 5 shows the NMR spectra of a- and c-Al_0.36_ST materials. Usually, there are two coordination environments for Al atoms in chalcogenide materials^24^,^25^: fourfold coordination Al(4) and sixfold coordination Al(6). The NMR chemical shifts corresponding to Al(4) and Al(6) vary between 59 ~ 62 and +9 to −6 ppm, respectively^25^. The fivefold coordination Al(5) is a transitional state between Al(4) and Al(6) with chemical shift peaking between them. The divided peak area ratio of Al(4):Al(6) is 85:15 for a-Al_0.36_ST as shown in Figure 5a, proving a majority of Al atoms being fourfold coordinated. Note that a-GST also has both fourfold and sixfold coordinated Ge atoms^26^. For c-Al_0.36_ST, in contrast, the area ratio of Al(4):Al(5):Al(6) is 21:5:74, as shown in Figure 5b. It agrees with our previous prediction that sixfold coordinated Al atoms shall prevail in c-Al_x_ST. We speculate the fivefold ones may also belong to the defective octahedrons. Like some tetrahedral Ge atoms found in c-GST^27^, fourfold Al atoms also exist in c-Al_x_ST. The tetrahedral Al atom utilizes its three valence electrons with one transfer electron from Te (Sb) to form covalent bonds upon *sp*^3^ hybridization^11^,^23^. It is reasonable to infer that nearly 65% of the total Al atoms undergo reversible tetrahedron-octahedron rearrangements during phase transition. Similar phenomenon was also observed in Al-As-Te glass that a larger fraction of fourfold coordinated Al atoms can promote memory switching over threshold switching^24^.

Figure 6 summarizes the evolution of representative local motifs during crystallization for a-GST and a-Al_0.36_ST. In a-GST, the Ge tetrahedron-to-octahedron reconfiguration is the key that triggers the nucleation and hence impacts the transition speed^28^. It has been established that the crystallization activation energy (*E_a_*) of a-GST is related to the reconfiguration of Ge-centered tetrahedrons to octahedrons^8^. Coincidentally, the *E_a_* of a-Al_0.36_ST (2.29 eV)^12^ is comparable to that of GST (2.24 eV)^29^. Indeed, as Al content further increases, the *E_a_* of a-Al-Sb-Te becomes larger (*E_a_* = 3.10 eV for a-Al_0.69_ST)^12^. Obviously like Ge in a-GST, most of the fourfold coordinated Al atoms in a-Al_x_ST have to overcome the bigger energy barrier to form sixfold coordinated Al in c-phase. The Al-centered octahedrons then may act as nucleation centers for surrounding Sb-centered motifs to align orderly.

We speculate that in other IIIA M-Sb-Te materials, the M-centered tetrahedron-to-octahedron reconfiguration may also be a decisive factor in determining their recrystallization procedures. Transforming such rigid M-centered motifs, in Ga-Sb-Te^15^ and In-Sb-Te^19^ etc., no doubt demands larger *E_a_*. That is why IIIA M-Sb-Te materials always show better data retention than that of GST. Because the lattice distortion and stress induced by cation M may lead to gradual phase segregation, M-Sb-Te materials usually show degenerated cyclabilities. Not surprisingly, there is no data claiming the better PCM endurance than that of the GST based device. Indeed, the wise way is to use IIIA M-Sb-Te PCM as a substitute for conventional NOR/NAND Flash memory which prefers good data retention, low power consumption, and fast response speed over cyclability.

In Summary, through experimental and theoretical studies, the Al-centered tetrahedron-octahedron rearrangements in phase transition of Al-Sb-Te system are described. We believe the reconfiguration of Al-centered motifs and its impact on surrounding Sb-centered motifs may simultaneously play the dominant roles to thermally stabilize the amorphous phase, reduce the amorphization energy, and affect the PCM device cyclability. We expect the performances of IIIA M-Sb-Te based PCM device to be further improved upon a thorough investigation of the physical nature of this material system.

## Methods

### Sample preparation and experimental details

The ST and Al_x_ST films in this work were prepared by physical vapor deposition method via co-sputtering pure Al and ST alloy targets at room temperature. The as-deposited Al_0.36_ST and Al_0.69_ST films are amorphous. The XRD tests were carried out on 500-nm-thick films deposited on the SiO_2_/Si substrates using PANalytical X'Pert PRO diffractometer with a Cu Kα (λ = 0.15418 nm) radiation source. A mass of ~20 mg Al_x_ST powder scratched from glass substrate was used for magic-angle-spinning (MAS) NMR measurements recorded with 9.4 and 16.4 T wide bore Bruker Avance III spectrometers equipped with a 3.2 mm HXY probehead (in double resonance mode). A MAS zirconia rotor with a spinning rate of 14 kHz was used to measure the spectra. Chemical shifts were recorded with respect to Al(NO_3_)_3_ aqueous solution as an external reference. All c-samples were obtained by annealing in N_2_ atmosphere to avoid oxidation.

### *Ab-initio* theoretical simulation

The theoretical investigations were performed by employing the *ab initio* calculations based on the density functional theory (DFT)^30^. The Vienna *Ab-initio* Simulations Package (VASP)^31^ was used for the first-principles calculations. The projector augmented wave (PAW)^32^ pseudopotentials were used to describe electron-ion interactions. For the exchange-correlation energies between electrons, the Perdew-Burke-Ernzerhof (PBE)^33^ function was employed. The energy cutoff was chosen to be 320 eV for all models and a 2-fs time step was used for the MD. The a-picture of Al_0.36_ST was obtained by melt-quench process. An initially c-structure containing 180 atoms was firstly melted at 2300 K, and then gradually quenched down to 1100 K (about 200 K above the melting temperature of ST^34^) at a quenching rate of −50 K ps^−1^. The liquid structure was further equilibrated for 9 ps at 1100 K. To generate the a-structure, the liquid structure was subsequently quenched to 300 K at a rate of −50 K ps^−1^. Finally, the system was retained at 300 K for 9 ps.

## Author Contributions

M.X. and X.L. performed the AIMD calculations. K.D. and F.R. prepared the film samples and carried out the XRD, NMR and so on. F.R. and X.L. carried out theoretical analysis and together wrote this paper with help from all co-authors. The project was initiated and conceptualized by F.R., L.W. and Z.S.

## Figures and Tables

**Figure 1:**
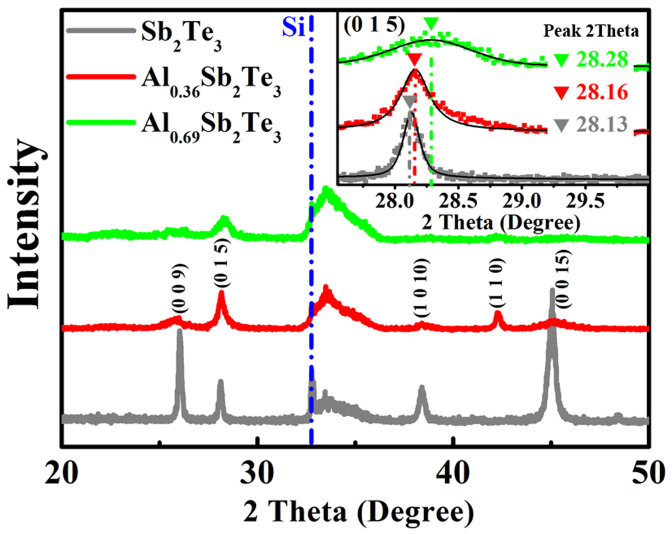
The X-ray diffraction (XRD) results of Al doped Sb_2_Te_3_. XRD curves of 300°C annealed Sb_2_Te_3_, Al_0.36_Sb_2_Te_3_, and Al_0.69_Sb_2_Te_3_ films on Si (1 0 0)/SiO_2_ substrates. Position change of peak (0 1 5) is shown enlarged in inset image, which suggests that the doped Al atoms enter the Sb_2_Te_3_ lattice and change the lattice parameters.

**Figure 2:**
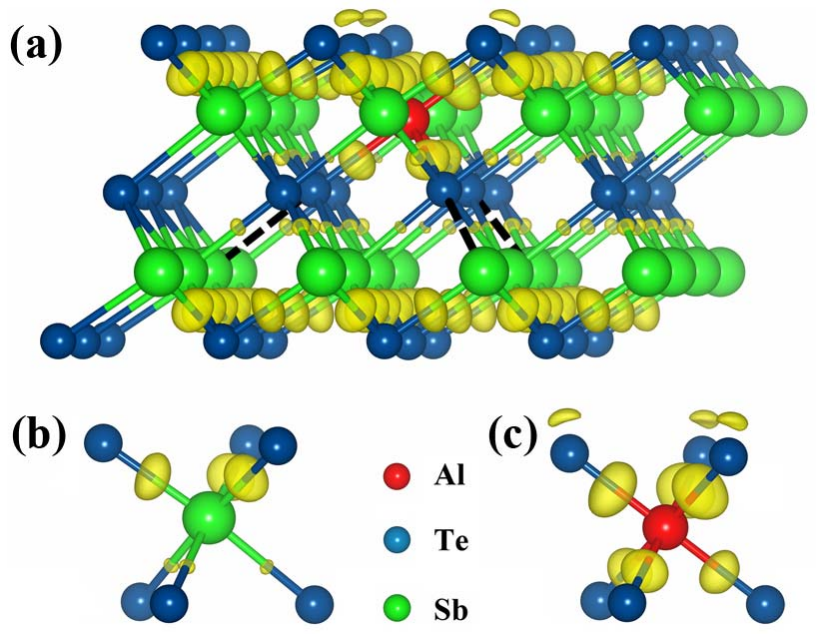
Bond chemistry of Al doped crystalline Sb_2_Te_3_. (a) Hexagonal lattice of Al doped Sb_2_Te_3_ shown by charge density difference. (b) and (c) are the Sb- and Al-centered octahedrons. The isosurface value is fixed at +0.005 e·a_0_^−3^ (a_0_ = bohr). The formation of six strong Al-Te bonds weakens three adjacent Te-Sb bonds in opposite bonding direction of Al-Te bonds [dashed lines in (a)].

**Figure 3:**
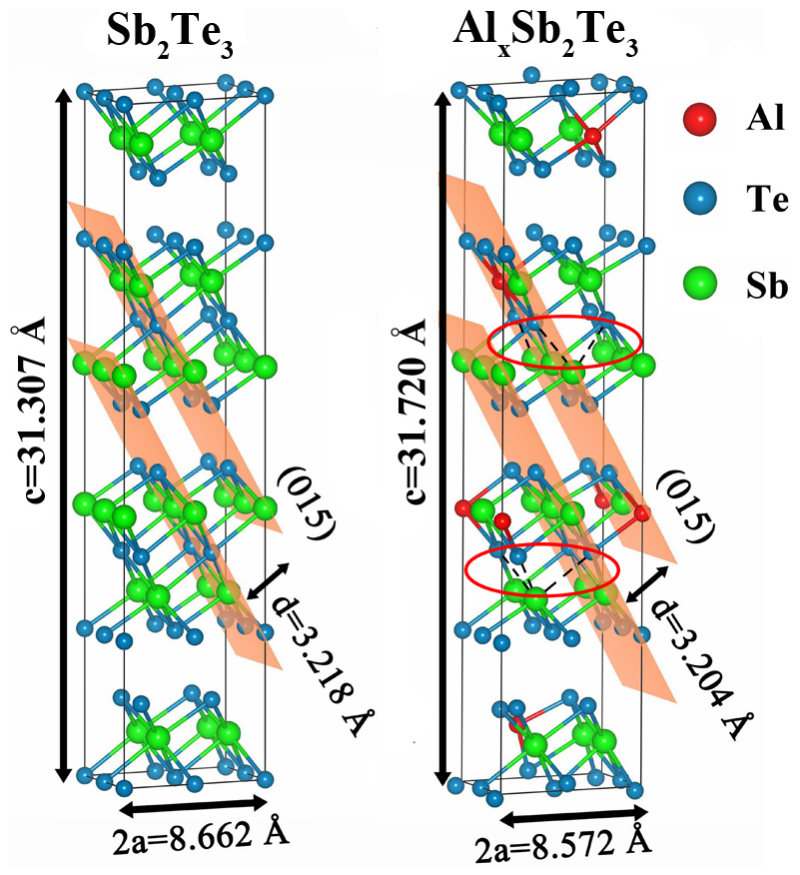
Lattice structures of crystalline Sb_2_Te_3_ and Al doped Sb_2_Te_3_. The crystalline structure of Sb_2_Te_3_ and Al doped Sb_2_Te_3_ are optimized and reveal differences in lattice constants (*a* and *c*) and the changed *d* spacing between (0 1 5) planes. Adjacent weakened Te-Sb bonds are also shown in dashed lines.

**Figure 4:**
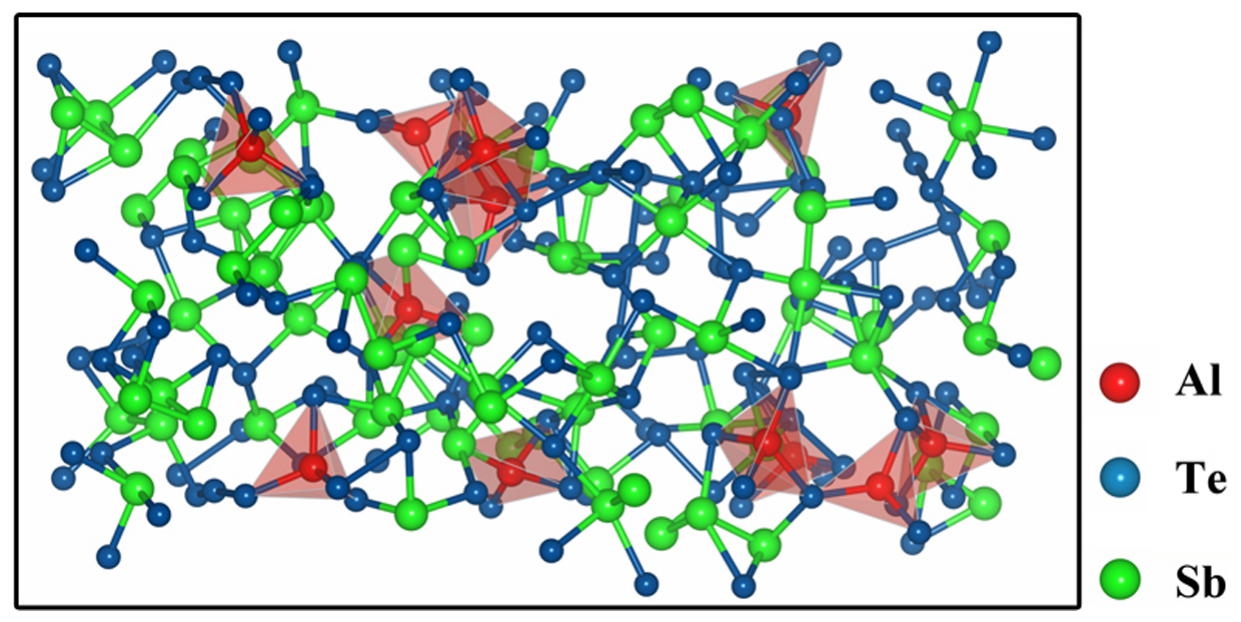
The Al-centered tetrahedrons in amorphous Al_0.36_Sb_2_Te_3_. The amorphous structure of Al_0.36_Sb_2_Te_3_ depicts that a majority of the Al are bonded with Te and fourfold coordinated in the tetrahedral-like geometry, which are highlighted as red polyhedrons.

**Figure 5:**
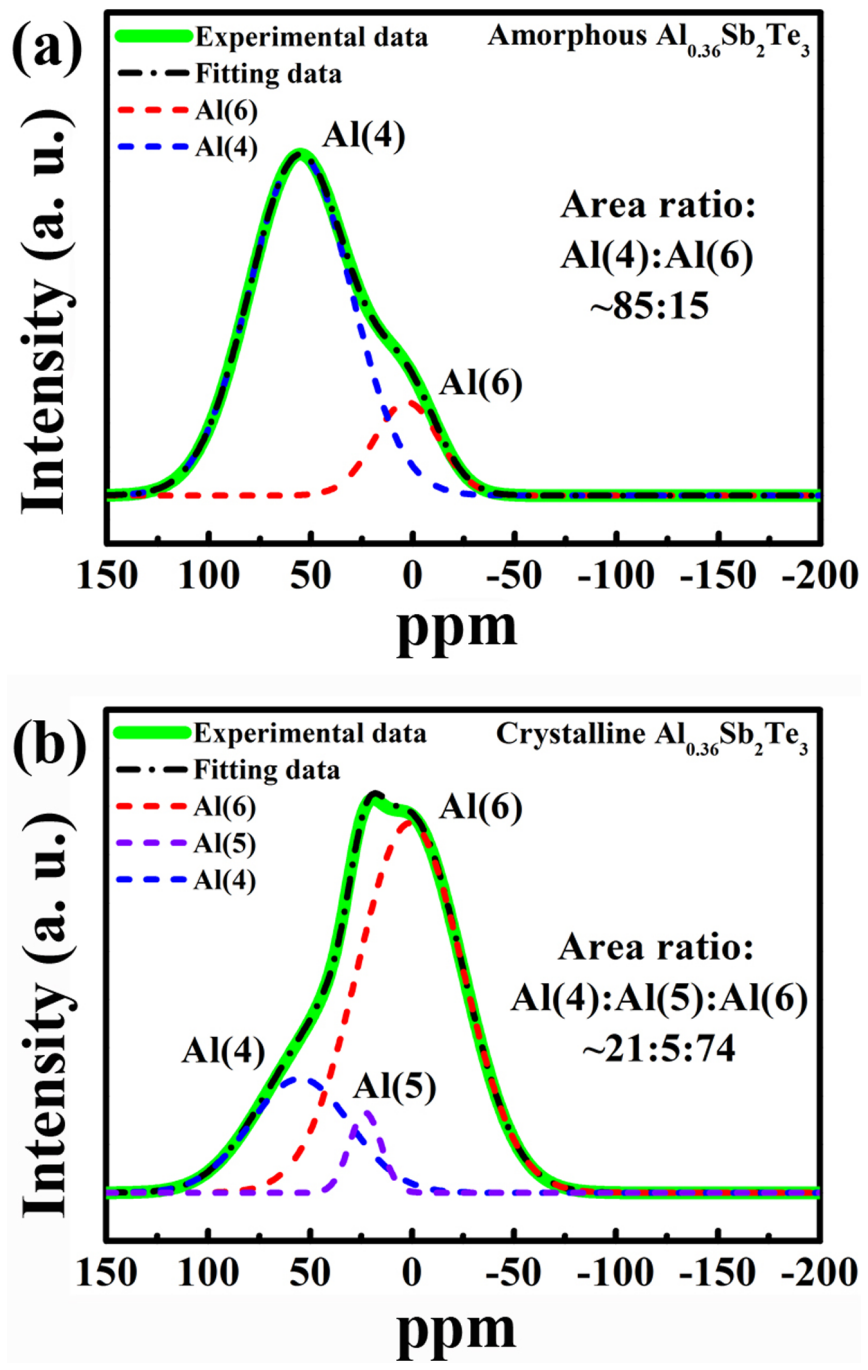
Nuclear magnetic resonance (NMR) results of Al_0.36_Sb_2_Te_3_ materials. ^27^Al magic-angle-spinning NMR spectra of (a) amorphous and (b) crystalline Al_0.36_Sb_2_Te_3_ materials with a spinning speed of 14 kHz. The chemical shifts near 0 and 60 ppm are assigned octahedral Al(6) and tetrahedral Al(4) coordinations, respectively. The results show a majority of Al atoms being fourfold coordinated in amorphous state while being sixfold coordinated in crystalline state.

**Figure 6:**
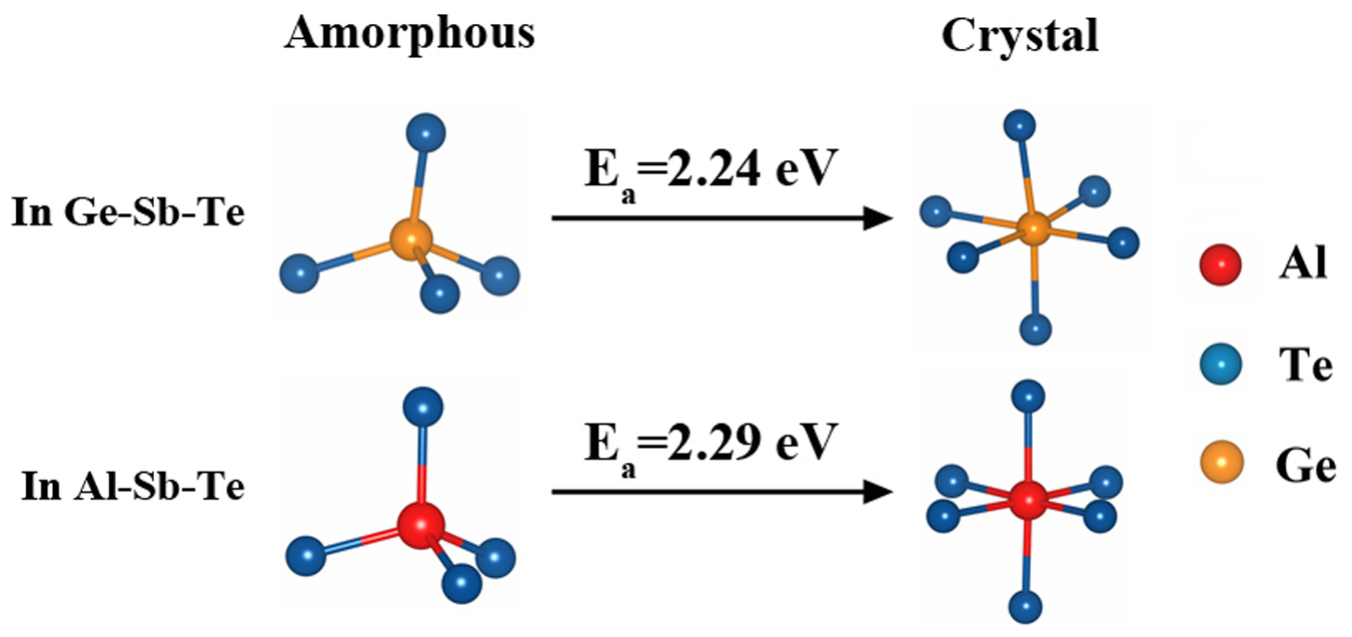
Nucleation processes for amorphous Ge_2_Sb_2_Te_5_ and Al_0.36_Sb_2_Te_3_. The representative structure evolutions of Ge- and Al-centered motifs during the nucleation of amorphous Ge_2_Sb_2_Te_5_ and Al_0.36_Sb_2_Te_3_ are shown and the activation energies (*E_a_*s) are also labeled. Similar to Ge, the Al atoms reveal tetrahedron-to-octahedron reconfiguration which triggers the nucleation and hence improves the thermal stability.

**Table 1:** Short and long bond lengths in optimized crystalline Al_0.36_Sb_2_Te_3_

	Al-Te	Sb-Te
Bond length (Å)	2.83, 2.92	3.03, 3.19

**Table 2:** The average coordination number and most possible bond angle for each element in amorphous Al_0.36_Sb_2_Te_3_

Al_0.36_Sb_2_Te_3_	Al	Sb	Te
Peak position of the bond angle distribution (°)	108	92	91
Average coordination number	4.08	3.74	2.82
